# The Enemy within: Propagation of Aberrant Corticostriatal Learning to Cortical Function in Parkinson’s Disease

**DOI:** 10.3389/fneur.2013.00134

**Published:** 2013-09-12

**Authors:** Jeff A. Beeler, Giselle Petzinger, Michael W. Jakowec

**Affiliations:** ^1^Department of Psychology, Queens College, City University of New York, New York, NY, USA; ^2^Department of Neurology, University of Southern California, Los Angeles, CA, USA; ^3^Division of Biokinesiology and Physical Therapy, University of Southern California, Los Angeles, CA, USA

**Keywords:** corticostriatal plasticity, striatopallidal pathway, dorsolateral striatum, cortical compensation, basal ganglia

## Abstract

Motor dysfunction in Parkinson’s disease is believed to arise primarily from pathophysiology in the dorsal striatum and its related corticostriatal and thalamostriatal circuits during progressive dopamine denervation. One function of these circuits is to provide a filter that selectively facilitates or inhibits cortical activity to optimize cortical processing, making motor responses rapid and efficient. Corticostriatal synaptic plasticity mediates the learning that underlies this performance-optimizing filter. Under dopamine denervation, corticostriatal plasticity is altered, resulting in aberrant learning that induces inappropriate basal ganglia filtering that impedes rather than optimizes cortical processing. Human imaging suggests that increased cortical activity may compensate for striatal dysfunction in PD patients. In this Perspective article, we consider how aberrant learning at corticostriatal synapses may impair cortical processing and learning and undermine potential cortical compensatory mechanisms. Blocking or remediating aberrant corticostriatal plasticity may protect cortical function and support cortical compensatory mechanisms mitigating the functional decline associated with progressive dopamine denervation.

As a primary site of dopamine denervation in Parkinson’s disease (PD) (1–3), pathophysiology in the dorsolateral striatum (DLS, equivalent to the posterior putamen in primates) is widely believed to play a central role in motor symptoms associated with disease progression. In the healthy brain, motor performance relies on the interaction between automatic (habit) and goal-directed (volitional) control of movement (4). Impairment in DLS function arising from denervation may induce degradation of automatic, implicit control of motor movements, and a compensatory shift to goal-directed, cortical control (4, 5). Accumulating imaging studies demonstrate that subjects with PD exhibit altered patterns of cortical activation [reviewed in Ref. (6, 7)]. Although pathophysiological changes related to cortical pathology may also contribute, it is believed that altered cortical activity reflects compensatory cortical circuit changes related to striatal dysfunction observed in early stages of disease progression (7–14). While the focus here is on cortical compensation, alterations in other circuit activity, including cerebellar circuits (15, 16), may also play an important compensatory role in striatal dysfunction.

In this brief perspective, we consider the relationship between the DLS and cortical function and outline a hypothesis suggesting that *aberrant corticostriatal plasticity under conditions of dopamine denervation actively degrades cortical processing*, gradually undermining cortical function and compensatory mechanisms. The theme of this special topic focuses on plasticity in sensorimotor circuitry involving the primary motor cortex, M1. The DLS and M1 are reciprocally modulated through re-entrant cortical basal ganglia-thalamo-cortical circuits (17, 18). The concepts described will refer to cortex and basal ganglia generally as our focus is the broader architectural and functional relationship between these two neural structures composing a circuit.

## The Dorsolateral Striatum: Optimizing Cortical Activity

As the primary input nucleus of the sensorimotor cortico-basal ganglia loop (19), the DLS of the basal ganglia contributes to motor learning and execution (20–24). Associated with stimulus-response learning, the DLS is believed to be a primary substrate for the development of automaticity associated with implicit, procedural learning, particularly sequencing, critical for the fluid execution of complex motor actions [reviewed in Ref. (25)]. M1 exhibits intrinsic activity-dependent synaptic plasticity, important for motor skill and sequence learning (26). This activity is shaped by both intracortical afferents and basal ganglia efferents (27).

**Figure 1:**
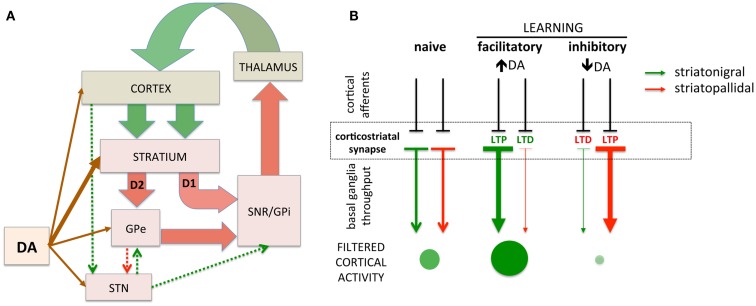
**Simplified schematic of cortico-basal ganglia circuitry and corticostriatal filtering of cortical activity**. **(A)** Dual corticostriatal architecture showing the direct and indirect pathways that express D1 and D2 dopamine receptors, respectively. Arrow colors reflect excitatory and inhibitory neurotransmitters. **(B)** Schematic diagramming selective facilitatory and inhibitory corticostriatal learning. LTP and LTD in the striatonigral and striatopallidal pathways, respectively (middle panel), facilitate cortical activity while the converse (LTD in striatonigral and LTP in striatopallidal) inhibit cortical activity. Green/red arrows represent striatonigral direct and striatopallidal indirect pathways, respectively. Direction of plasticity (LTP vs. LTD) colored red/green to indicate functional facilitation/inhibition of cortical throughput. The size/intensity of green circles represent the increase/decrease in activity of a synapse-specific cortical afferent induced by basal ganglia modulation.

The basal ganglia are comprised of GABAergic medium spiny neurons (MSNs) of the striatonigral (direct) and striatopallidal (indirect) projections. These striatal projections modulate inhibition of cortical activity through the output nuclei of the basal ganglia that provide tonic inhibition of excitatory thalamocortical projections (17, 18). In turn, cortical glutamatergic afferents on striatal MSNs can activate the striatonigral or striatopallidal pathways, disinhibiting or inhibiting cortical activity, respectively [(28, 29); Figure 1A]. The responsiveness of striatonigral and striatopallidal MSNs to specific cortical synaptic inputs can be modulated through synaptic strength (Figure 1B). Increased synaptic strength is commonly defined by the emergence of long-term potentiation (LTP) and decreased synaptic strength by long-term depression (LTD). For example, a specific cortical synaptic input may be potentiated (LTP) at striatonigral disinhibitory MSNs while a cortical synaptic input on striatopallidal inhibitory MSNs is depressed (LTD), leading to the combined facilitation of cortical activity (30–34). Increasing evidence supports bidirectional plasticity at corticostriatal synapses (35–41), suggesting the opposite pattern can arise as well. For example, a specific cortical synaptic input might be potentiated, increasing striatopallidal MSN activity in response to that input and increasing cortical inhibition. The basal ganglia, through selective synaptic potentiation and depression, facilitate particular cortical activities while inhibiting others, thus filtering cortical activity through cortico-basal ganglia-thalamo-cortical loops. While the precise function of this filter remains subject to debate, it is hypothesized that the purpose of this basal ganglia filter is to select one action and suppress others (29, 42). An alternative view is that the basal ganglia, by selectively facilitating productive, task-relevant cortical activity and suppressing non-productive and/or irrelevant activity, optimizes cortical processing to increase speed and efficiency.

### Dopamine regulates cortical filtering through the basal ganglia

**Figure 2:**
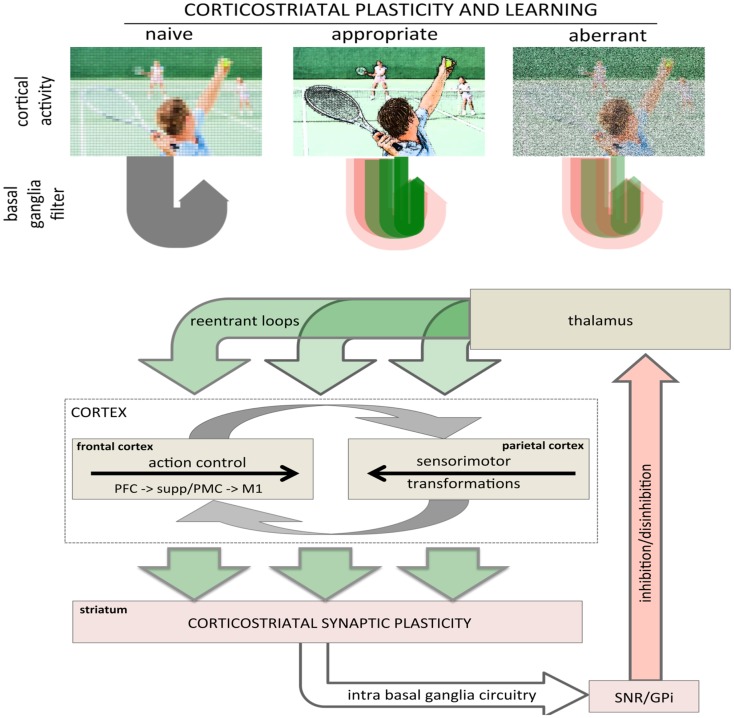
**Role of corticostriatal plasticity and learning in basal ganglia filtering of cortical activity**. (top) Conceptual illustration of basal ganglia filtering of cortical activity through corticostriatal plasticity and learning. The naïve, pre-learning state is represented in the left panel and processing through the basal ganglia is undifferentiated (gray arrow loop). After appropriate corticostriatal learning (middle panel), task-related elements are sharpened and highlighted (racquet, ball, arm, other players), represented in the corticostriatal loop as a combination of facilitation and inhibition with strong, task-relevant facilitation (sharp, dark green arrow loop). Under aberrant learning (right panel), inappropriate LTP in the inhibitory striatopallidal pathway induces inappropriate inhibition (red shaded arrow loops) and diminished facilitation (green shaded arrow loops) in the corticostriatal filter causing task-related elements become increasingly noisy and less distinct against background compared even to naïve processing (left panel). (bottom) Schematic showing rudimentary architecture for basal ganglia filter of cortical activity highlighting the general loop architecture. Large green arrows represent cortical inputs to the striatum and re-entrant projections returning to the cortex via the thalamus. The intrabasal ganglia circuitry has been collapsed to highlight the basic loop architecture. The cortical schematic has been expanded to represent the two primary intracortical information flows mediating action selection and motor control. The left cortical box represents traditional frontal motor control where information flows rostral to caudal from the prefrontal cortex to M1. The right cortical box represents parietal processing where information flows caudal to rostral mediating sensorimotor transformations specifying movements. These two are intricately interconnected, represented by reciprocal gray arrows. Image used in top panel licensed from Polka Dot Images/Thinkstock.

Bidirectional corticostriatal plasticity is regulated by dopamine (31, 33, 34, 37, 43), widely believed to encode positive and negative reward prediction errors (RPEs) by firing bursts of action potentials or pausing tonic activity, respectively (44, 45). This RPE provides a teaching signal indicating positive and negative (better or worse than expected) outcomes. By regulating bidirectional corticostriatal synaptic plasticity, a dopamine-mediated teaching signal selectively enhances or diminishes cortical inputs associated with positive and negative outcomes, respectively. Through dopamine regulation, corticostriatal plasticity can selectively filter and highlight cortical activity determined to be relevant and beneficial (Figure 2, top). The net result is that cortical activity that is productive and yields a positive outcome will be selectively processed and amplified to complete a motor task.

## Aberrant Plasticity and Learning: Inverting Optimization

Increasing evidence suggest that reduced dopamine may shift corticostriatal plasticity in striatopallidal synapses favoring LTP rather than LTD, inverting plasticity such that conditions that would normally yield LTD produce LTP instead (38–41). The net effect of this would be that everywhere a cortical afferent should be disinhibited it would, instead, be *further inhibited*. Such a shift or inversion in the directional control of plasticity – the aberrant learning hypothesis – would transform an optimizing substrate into an “anti-optimizing” one that impedes rather than facilitates responding (25, 41, 46–48). Aberrant learning, then, transforms the basal ganglia that normally functions to filter and facilitate cortical activity into a disruptive filter that impedes motor activity.

Aberrant learning arising from reduced striatal dopamine contributes significantly to impaired motor performance over and above the direct motor effects of diminished dopamine (41, 48). The correction of aberrant learning and abnormal corticostriatal plasticity may represent an important component of L-DOPA treatment in PD and underlie the poorly understood long-duration response (LDR), where the benefits of dopamine replacement on motor performance persists beyond the pharmacokinetic half life of L-DOPA (25, 41, 48–50). Given the number of potential modulators of synaptic plasticity within the striatum, that include but are not limited to adenosine, glutamatergic and cholinergic neurotransmission, decreasing inappropriate potentiation at corticostriatal synapses in the striatopallidal pathway may serve as an important therapeutic target for facilitating motor learning and recovery of function in PD. For example, correction of aberrant learning may be an important therapeutic mechanism of adenosine antagonists (36, 41).

The aberrant learning hypothesis can be understood as an extension of the classic model of PD where there is an imbalance between the direct and indirect pathways (28, 29, 51–53). With aberrant learning, this imbalance is *structurally encoded* as inappropriate synaptic strengths [i.e., inverted corticostriatal plasticity; (25, 41, 46–48, 54)]. Thus, even if dopamine is restored, the inappropriate learning that has already been established will continue to degrade motor performance until appropriate synaptic plasticity and learning has replaced the inappropriate (41). Conversely, if dopamine is reduced (e.g., discontinuation of L-DOPA), performance will initially be partially protected as the appropriate synaptic strengths will facilitate corticostriatal throughput; however, as aberrant plasticity and learning return, synaptic structure will again become inverted and anti-optimal and initially retained function will deteriorate (48).

## Reciprocal Relationship between Cortical and Striatal Plasticity and Learning

Learning is the encoding of information through alterations in synaptic strengths that underlie memory formation and skill acquisition. Though the cortex and striatum both exhibit learning and synaptic plasticity, how learning in each substrate affects learning in the other is poorly understood.

### Cortical learning shapes basal ganglia activity and learning

As the cortex is a primary afferent to the striatum, alterations in synaptic plasticity that occurs in the cortex will change the afferent input to the basal ganglia, affecting both information flow through the basal ganglia and activity-dependent corticostriatal synaptic plasticity. For example, if the cortex learns to enhance the activity of one ensemble of neurons and diminish the activity of others, this differential will be reflected in the pattern of MSNs activation and the altered synaptic inputs may induce changes in corticostriatal synaptic strength. In this case, alterations in synaptic plasticity of the cortex may directly influence downstream synaptic plasticity within the striatum.

### Striatal learning shapes cortical activity and learning

Though cortical activity and learning shape activity in the striatum, evidence suggests that the striatum may learn earlier and faster and may shape cortical learning (55), consistent with the idea that the basal ganglia provide a filter for cortical activity. By selectively altering cortico-basal ganglia throughput of specific cortical afferents, corticostriatal plasticity shapes cortical activity; by modulating cortical activity, the basal ganglia can influence activity-dependent cortical learning.

### Reciprocal learning provides a gain mechanism for learning

Cortical activity that is facilitated through the basal ganglia filter will be more likely to undergo activity-dependent, Hebbian synaptic plasticity, strengthening those particular patterns of cortical activity in the future. Conversely, cortical activity inhibited by the basal ganglia will have lower probability of activity-dependent plasticity (or favor synaptic depression), diminishing that activity in the future. In turn, this altered pattern of cortical afferents to the striatum will differently activate MSNs and further modify corticostriatal synaptic strengths, which further modifies cortical activity and learning.

## The Domino Effect: Aberrant Learning and Disrupted Cortical Filtering

Reduction in cortical activity in cortical regions that participate in the dorsal striatal sensorimotor loop (e.g., M1, PMC, posterior parietal) as well as reduced activity in regions associated with the cognitive loop (e.g., DLPFC) have been well documented in PD (6, 56). Reduced cortical activity in these regions is consistent with the classic model of an imbalance between the direct and indirect pathway that results in increased inhibitory tone on cortical activity. However, numerous studies have observed increased activity in these same regions associated with task performance (8–13, 15, 16, 57–62). While task-related increases in cortical activity may be construed as compensatory, it may reflect reduced processing efficiency. With the acquisition of automaticity, healthy normal controls exhibit *decreased* cortical activity while PD subjects do not show such reductions, consistent with increased cortical load arising from dysfunction in corticostriatal circuits (58). Such increased cortical activity may arise from the loss of appropriate corticostriatal filtering. In a recent study, Ng et al. (57) have shown spatially greater cortical activation (i.e., more spread out) in unmedicated PD subjects performing a simple motor task compared to healthy controls; this spatially greater activation is normalized by L-DOPA, which the authors characterize as a “focusing effect.” In the view adopted here, the increased cortical activity could reflect both compensatory cortical circuits and pathophysiology in the basal ganglia filter. First, denervation in the DLS induces greater inhibitory tone, diminishing cortical activity, which in turn leads to compensatory functional connectivity that increases intracortical and cerebellar drive on cortical activity. Second, this increased activity lacks the filter that “focuses” cortical activity, resulting in overall greater, less efficient cortical activation. The net result is that the cortex has to work harder to maintain behavior and does so with less efficiency.

## Propagation of Aberrant Learning

As functional connectivity shifts to drive cortical activity and maintain behavior, cortical mechanisms of activity-dependent synaptic plasticity continue to facilitate learning to refine and calibrate cortical networks and ensembles to adapt to new patterns of afferent inputs. In effect, the cortex has to learn the adaptations. If the filtering or “focusing” effect of the DLS and the sensorimotor basal ganglia loop were merely absent, learning might simply be slower and less efficient. From the perspective of the aberrant learning hypothesis, however, the problem is more insidious. Dopamine denervation induces inverted corticostriatal plasticity in the striatopallidal pathway that actively inhibits precisely those high activity cortical afferents that should be facilitated, providing an anti-optimizing filter. Instead of increasing signal-to-noise ratio – enhancing productive and diminishing non-productive/irrelevant cortical activity – aberrant learning would actively diminish *signal*. Thus, as activity-dependent cortical learning adjust cortical synaptic strengths to improve performance and adapt to shifted functional connectivity, the resulting changes in cortical activity are subsequently opposed by aberrant learning in the DLS effectively filtering out such learning by inappropriately inhibiting precisely those activities the cortex has just learned to enhance. It is not difficult to imagine a progression where inappropriate filtering in conjunction with continued aberrant learning leads to further compensatory cortical activity that is less efficient and, in turn, also degraded by aberrant learning resulting in a vicious cycle of increasingly greater but less efficient cortical activity to maintain motor function.

## Clinical Implications and Further Research

Aberrant corticostriatal plasticity, from the perspective of the aberrant learning hypothesis, represents more than a simple loss of function. Rather, it is an active, insidious process that hijacks corticostriatal plasticity, not only impeding the role of the basal ganglia as an effective filter of cortical activity, but structurally encoding inappropriate learning as synaptic strengths that can actively disrupt cortical function. Such aberrant learning will gradually unravel and invert a lifetime of learning – and the millions of finely calibrated synaptic strengths that support that learning – accelerating functional deterioration associated with neurodegeneration.

As dopamine denervation progresses, cortical compensatory activity is engaged. The reciprocal relationship between cortical and basal ganglia structures, however, suggests that aberrant corticostriatal plasticity may increase the burden placed on the cortex while simultaneously interfering with its compensatory capacity. If true, this means that therapeutic agents that ameliorate aberrant learning would have the additional advantage of supporting cortical compensation. In particular, decreasing an inappropriate, “anti-optimizing” basal ganglia filter may facilitate improved cortical learning and adaptation. Therapeutic strategies specifically targeted at correcting or blocking aberrant learning may slow functional deterioration associated with neurodegeneration as well as support adaptive, compensatory mechanisms, providing a form of disease-modifying neuroprotection that may mitigate the functional decline associated with disease progression. Thus, targeting subcellular signaling pathways and cellular mechanisms specific to striatopallidal synaptic plasticity to either diminish inappropriate LTP or enhance appropriate LTD may offer an avenue for the development of alternative disease-modifying, neuroprotective therapeutics (25, 41, 50).

An important aspect of aberrant learning is that it introduces a delay between the pathophysiology (or its correction) and the resulting functional effects on behavior. That is, aberrant learning that occurs *now* will affect performance *in the future*. Conversely, improvement associated with corrected aberrant learning will be observed gradually over time as restored corticostriatal plasticity *relearns* and recalibrates synapses. It is precisely this delay in observing the effects of corrected aberrant learning that we have proposed underlies the LDR to L-DOPA (25, 48). If aberrant corticostriatal plasticity degrades cortical learning, a delay might be expected in the correction of impaired cortical functions as the cortex *also* undergoes relearning. Interestingly, it has been observed that several cognitive symptoms of PD do not appear to be improved with L-DOPA administration [e.g., Ref. (63)]. Why this might be is unclear, but one possibility is that cognitive symptoms associated with cortical functions may, at least partially, arise from impaired cortical learning *induced* by aberrant corticostriatal plasticity. If so, L-DOPA would not immediately correct previously degraded or inappropriate cortical learning; however, similar to the LDR, such symptoms might improve gradually over time as cortical learning is protected from the deleterious effects of aberrant corticostriatal plasticity. Conversely, Kishore et al. (64, 65) have recently observed a deficit in plasticity in M1 in newly diagnosed, untreated patients that is corrected under optimal medication (absent fluctuations or dyskinesia). This corrected plasticity is sustained even during the OFF state, suggesting it may represent a LDR (64). The degree to which these findings relate to dopamine replacement and a potential LDR action *directly* in M1 versus arising secondary to the correction of aberrant corticostriatal plasticity and its effects on cortical processing and learning remain to be determined.

This hypothesis has potentially significant implication for rehabilitative treatments in PD. Rehabilitation fundamentally involves repetition, i.e., practice, and is premised on mechanisms of learning and plasticity. If those underlying mechanisms are not only impaired, but potentially induce counter-productive, aberrant learning and plasticity, the efficacy of rehabilitation could be compromised. Rehabilitative strategies might be most beneficial, then, when conducted under optimal medication to maximally support cortical compensatory learning. The success of physical therapy programs, including programs which patients practice independently (eg., Big and Loud), may be contingent on training and practice being performed during the ON medication state.

In conclusion, agents that target and ameliorate aberrant corticostriatal plasticity in the striatopallidal pathway may represent an important avenue for the development of new therapeutics, potentially yielding a protective LDR-like treatment independent of dopamine replacement with its attendant complications. Remediating an insidious process of inappropriate corticostriatal synaptic plasticity may mitigate circuit deterioration and support cortical compensatory mechanisms, modifying the rate and severity of functional decline associated with disease progression. Further research on aberrant learning and its potential effects on cortical function and learning are needed and may yield new insights and treatment strategies.

## Conflict of Interest Statement

The authors declare that the research was conducted in the absence of any commercial or financial relationships that could be construed as a potential conflict of interest.
